# Supplementary material for: Nuclear position and local acetyl-CoA production regulate chromatin state
Source: Nature. 2024 Jun 5;630(8016):466–74. doi: 10.1038/s41586-024-07471-4 (PMC11168921; doi:10.1038/s41586-024-07471-4)
Supplement: Supplementary file 1 — This file contains Supplementary Data 1–3 and Supplementary Tables 1–6. [file 41586_2024_7471_MOESM1_ESM.pdf]

nature portfolio

https://doi.org/10.1038/s41586-024-07471-4

Supplementary information

Nuclear position and local acetyl-CoA production regulate chromatin state

In the format provided by the
authors and unedited

Supplementary Data 1

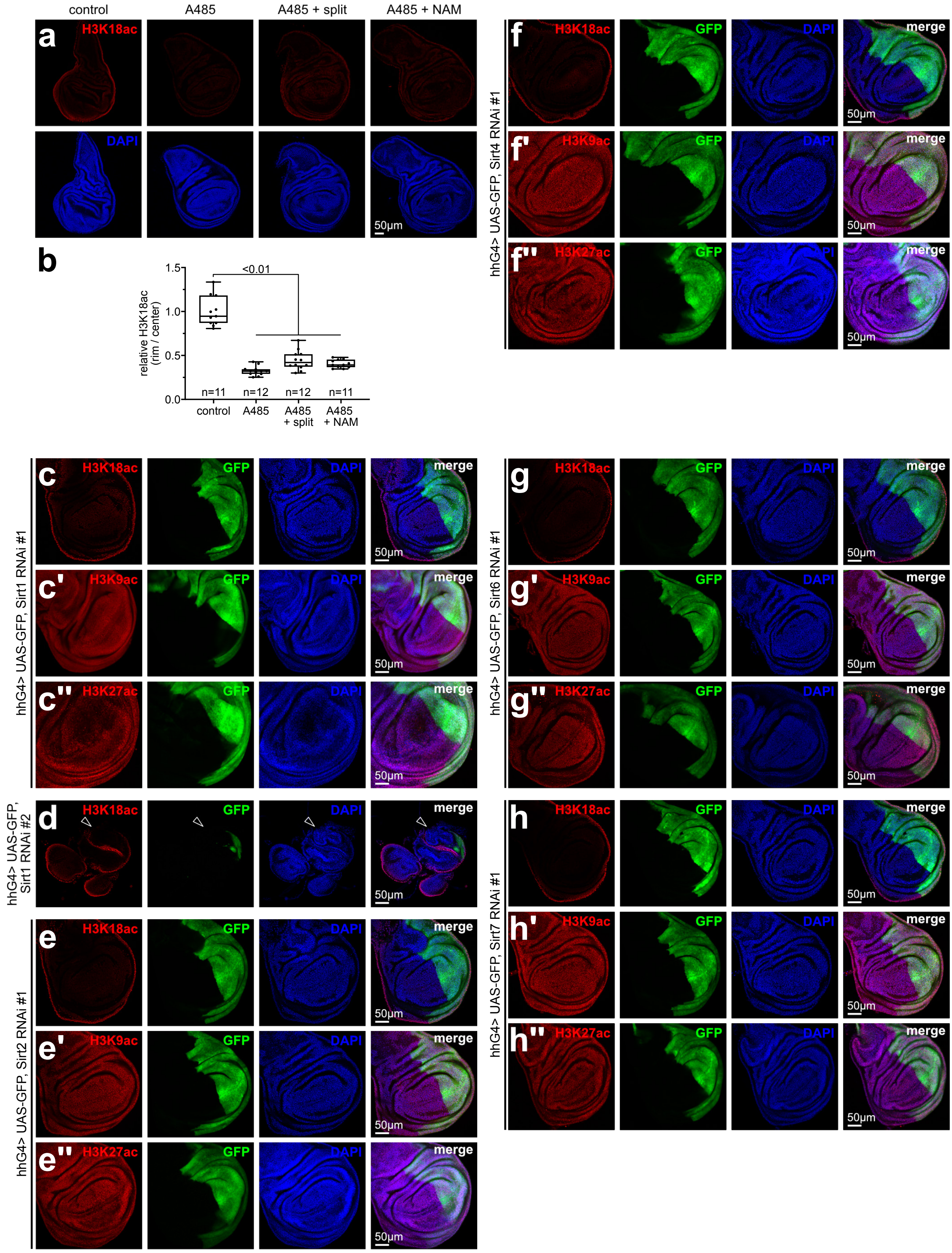

Supplementary Data 1: Screening data - effect of Sirtuin knockdowns on histone acetylation

(a-b) H3K18ac is not deacetylated by sirtuins (class III deacetylases) as pharmacological inhibition with splitomycin (split, 50 µM) or nicotinamide (NAM, 20 mM) does not prevent loss of H3K18ac upon nejire (nej) inhibition by A485 (20 µM). Discs incubated for 2 h in explant cultures with indicated inhibitors. Representative images in (a), quantified in (b). Statistical significance by Kruskal-Wallis test with Dunn's multiple comparisons test. (n=11-12 discs). Whisker plots: center line (median), box limits (1st and 3rd quartiles), whiskers (outer data points).

(c-h) Knockdown of Sirt1 (c-d), Sirt2 (e-e''), Sirt4 (f-f''), Sirt6 (g-g''), or Sirt7 (h-h'')) does not impact H3K18ac, H3K9ac, or H3K27ac levels in the posterior compartment (GFP+) indicating that these sirtuins are not responsible for deacetylation of these histone marks. Knockdown of Sirt1 with RNAi line #2 (d) caused a strong decrease in posterior compartment size. The wing disc is indicated by the open arrowhead. (c-c'', n=9 discs; d, n=7 discs; e-e'', n=9 discs; f-f'', n=9 discs; g-g'', n=9-11 discs; h-h'', n=9 discs).

Supplementary Data 2

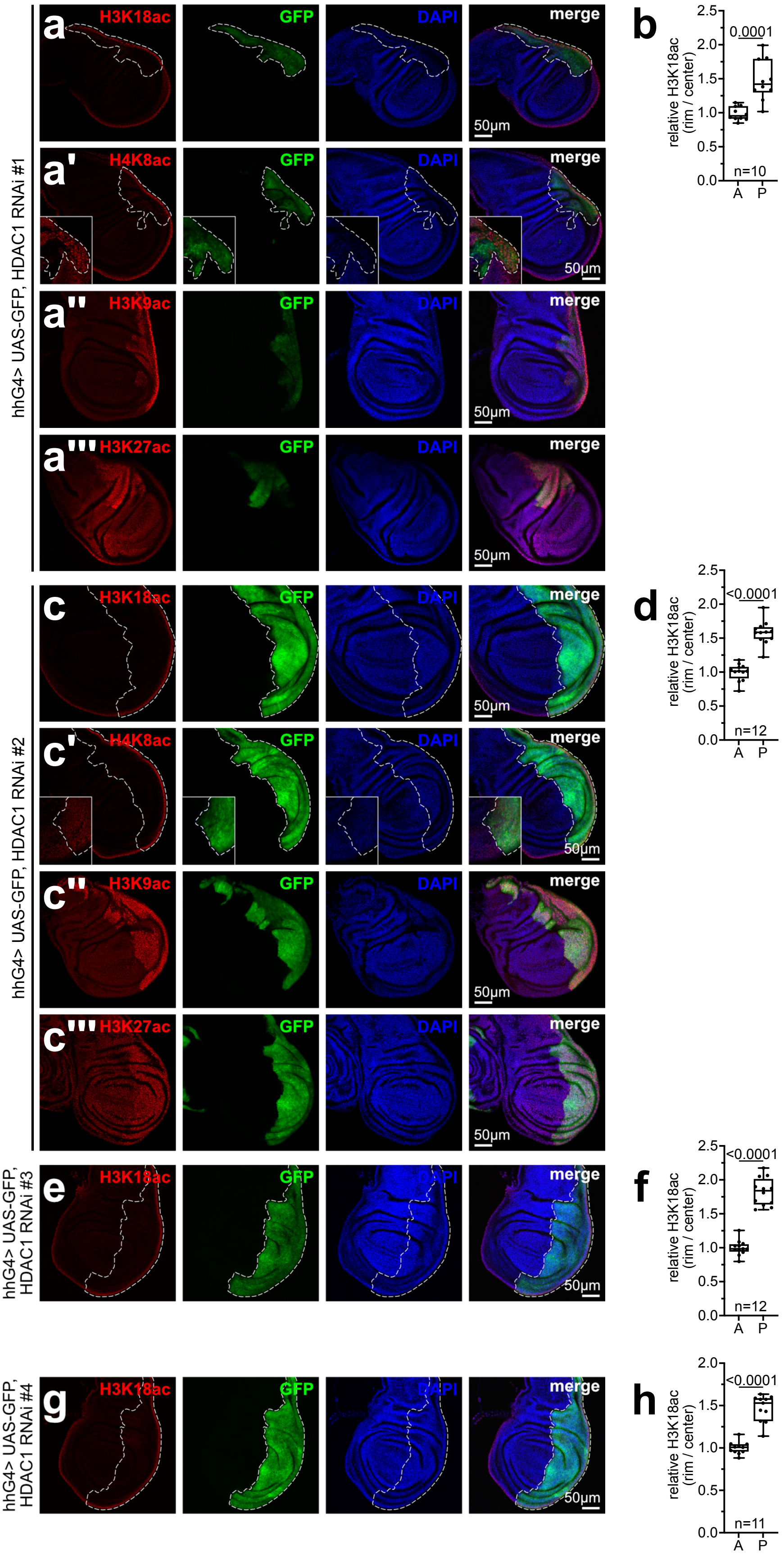

Supplementary Data 2: Effects of HDAC1 knockdown on various histone acetylation marks

(a-b) Knockdown of Histone deacetylase 1 (HDAC1) in the posterior compartment (GFP+) increases acetylation of H3K18 (a) and H4K8 (a') mildly, and increases acetylation of H3K9 (a'') and H3K27 (a''') strongly. The dashed line outlines the posterior compartment (GFP+). Inset in (a') shows a different x-y section from the same disc. Representative images of H3K18ac in (a), quantified in (b). Statistical significance by Mann-Whitney test (two-sided). 1 outlier was removed as determined by ROUT. (a-a''', n=10-16 discs; b, n=10-11 discs).

(c-d) Knockdown of HDAC1 with a second independent RNAi line in the posterior compartment (GFP+) causes a mild increase in acetylation of H3K18 (c), H4K8 (c'), and a clear increase in acetylation of H3K9 (c'') and H3K27ac (c'''). The dashed line outlines the posterior compartment (GFP+). Inset in (c') shows a different x-y section from the same disc. Representative images of H3K18ac in (c), quantified in (d). Statistical significance by Mann-Whitney test (two-sided). (c-c''', n=10-14 discs; d, n=12 discs).

(e-f) Knockdown of HDAC1 with a third independent RNAi line in the posterior compartment (GFP+) causes a mild increase in acetylation of H3K18 (e). The dashed line outlines the posterior compartment (GFP+). Representative images in (e), quantified in (f). Statistical significance by Mann-Whitney test (two-sided). (n=12 discs).

(g-h) Knockdown of HDAC1 with a fourth independent RNAi line in the posterior compartment (GFP+) causes a mild increase in acetylation of H3K18 (g). The dashed line outlines the posterior compartment (GFP+). Representative images in (g),

quantified in (h). Statistical significance by Mann-Whitney test (two-sided). (n=11 discs).

Whisker plots: center line (median), box limits (1st and 3rd quartiles), whiskers (outer data points). A, anterior; P, posterior

Supplementary Data 3

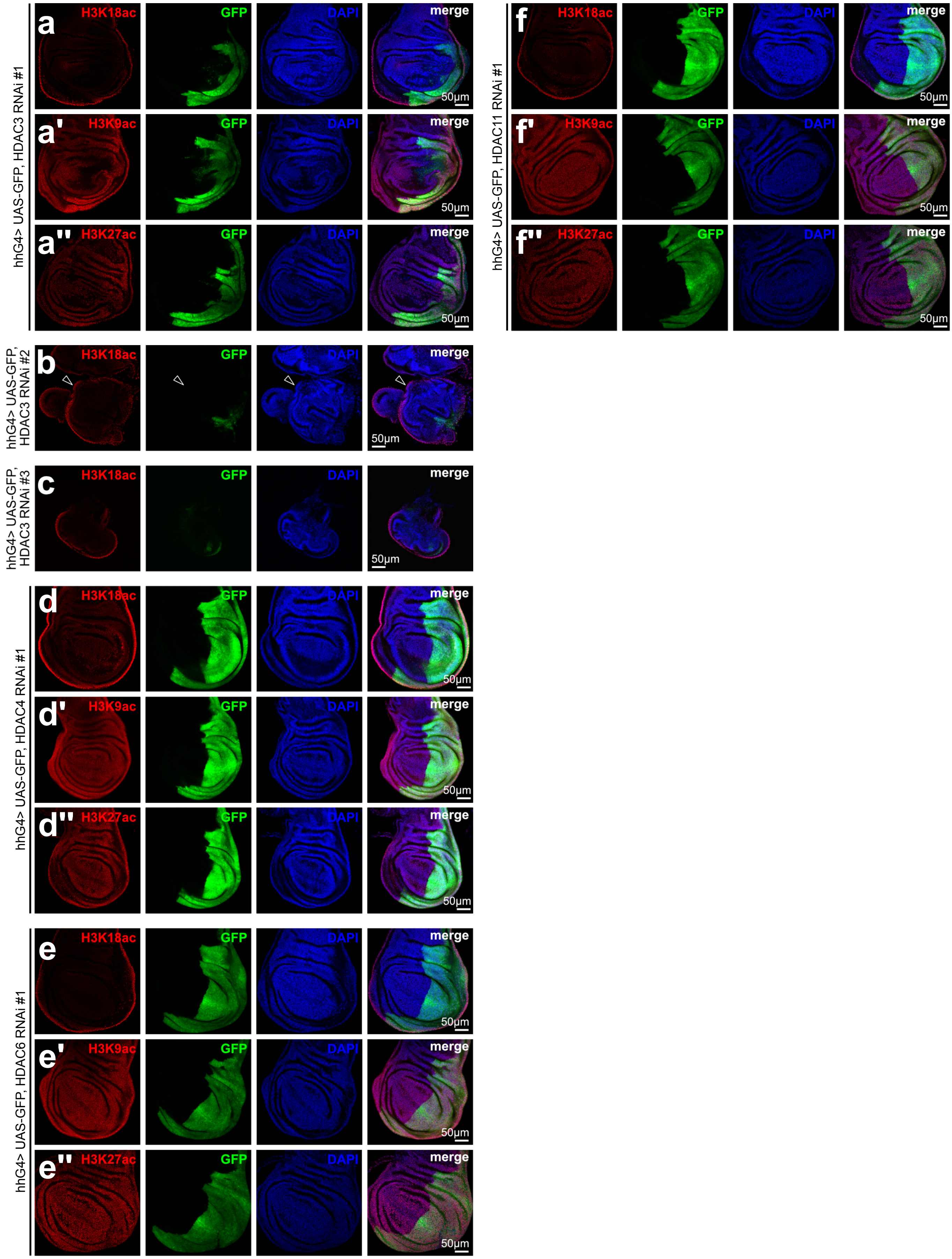

Supplemental Data 3: Effects of HDAC3, 4, 6, and 11 knockdowns on various histone acetylation marks

(a-a'') Knockdown of Histone deacetylase 3 (HDAC3) in the posterior compartment GFP+) increases H3K9ac (a') but not H3K18ac (a) or H3K27ac (a''). (n=10 discs).

(b-c) Knockdown of HDAC3 with a second and third independent RNAi line in the posterior compartment (GFP+) does not affect acetylation of H3K18 but caused a strong decrease in posterior compartment size. The wing disc in (b) is indicated by an open arrowhead. (b, n=5 discs; c, n=13 discs).

(d-f) Knockdown of HDAC4 (d-d''), HDAC6 (e-e''), or HDAC11 (f-f'') in the posterior compartment (GFP+) does not impact levels of H3K18ac, H3K9ac, or H3K27ac, indicating that these HDACs are not responsible for deacetylating these histone marks. (d-d'', n=11 discs; e-e'', n=9 discs; f-f'', n=9-10 discs).

Suppl Table 1: Effect of histone acetyltransferase or deacetylase knockdowns on different histone acetylation marks

Abbreviations: Elp3, Elongator complex protein 3; Gcn5, General Control Non-repressed protein 5; HDAC, histone deacetylase; nej, nejire; Sirt, sirtuin (n=5-16 discs)

gene	RNAi line	#	H3K18ac	H4K8ac	H3K9ac	H3K27ac	morphology
nej	KK102885	1					
Gcn5	KK108943	1					
Elp3	KK106128	1					
Sirt1	KK105502	1					
Sirt1	B53697	2					
Sirt1	B31636	3					
Sirt1	B32481	4					
Sirt1	KKK108241	5					
Sirt2	BKK103790	1					
Sirt2	B36868	2					
Sirt2	B31613	3					
Sirt4	KK110639	1					
Sirt4	B31638	2					
Sirt4	B36588	3					
Sirt4	B33984	4					
Sirt6	B34530	1					
Sirt6	B31399	2					
Sirt6	B36801	3					
Sirt7	B32483	1					
Sirt7	B31093	2					
Sirt7	B36802	3					
HDAC1	B33725	1					
HDAC1	B34846	2					
HDAC1	B31616	3					
HDAC1	B36800	4					
HDAC3	KK107073	1					
HDAC3	B64476	2					
HDAC3	B34778	3					
HDAC3	B31633	4					
HDAC4	B34774	1					
HDAC4	B28549	2					
HDAC6	KK108831	1					
HDAC6	B31053	2					
HDAC6	B34072	3					
HDAC11	KK108098	1					
HDAC11	B32480	2					

- mild/strong decrease
- weak increase
- no change
- weak decrease
- mild/strong decrease
- not tested

Suppl. Table 2: List of fly lines

Numbering of the RNAi lines (#) used in this paper is indicated.

Abbreviations: AcCoAS, acetyl-CoA synthase; AFG, actin flip-out Gal4; ATPCL, ATP citrate lyase; Elp3, Elongator complex protein 3

Gcn5, General Control Non-repressed protein 5; HDAC, histone deacetylase; hh, hedgehog; hsFLP, heat-shock flipase

IFP, infra-red protein; koi, klaroid; nej, nejire; PDH a, pyruvate dehydrogenase α; PDK, pyruvate dehydrogenase kinase; Rok, Rho kinase; Sirt, sirtuin

fly line	# in the paper	source	stock number
ATPCL RNAi	1	Bloomington	65175
Elp3 RNAi	1	VDRC	KK106128
Gcn5 RNAi	1	VDRC	KK108943
HDAC1 RNAi	1	Bloomington	33725
HDAC1 RNAi	2	Bloomington	34846
HDAC1 RNAi	3	Bloomington	31616
HDAC1 RNAi	4	Bloomington	36800
HDAC3 RNAi	1	VDRC	KK107073
HDAC3 RNAi	2	Bloomington	64476
HDAC3 RNAi	3	Bloomington	34778
HDAC3 RNAi	4	Bloomington	31633
HDAC4 RNAi	1	Bloomington	28549
HDAC4 RNAi	2	Bloomington	34774
HDAC6 RNAi	1	VDRC	KK108831
HDAC6 RNAi	2	Bloomington	31053
HDAC6 RNAi	3	Bloomington	34072
HDAC11 RNAi	1	VDRC	KK108098
HDAC11 RNAi	2	Bloomington	32480
koi RNAi	1	VDRC	KK108236
nej RNAi	1	VDRC	KK102885
nej RNAi	2	VDRC	KK105115
PDHa RNAi	1	VDRC	KK107209
PDK RNAi	1	VDRC	KK106641
Rok RNAi	1	VDRC	KK104675
Sirt1 RNAi	1	VDRC	KK105502
Sirt1 RNAi	2	Bloomington	53697
Sirt1 RNAi	3	Bloomington	31636
Sirt1 RNAi	4	Bloomington	32481
Sirt1 RNAi	5	VDRC	KKK108241
Sirt2 RNAi	1	VDRC	KK103790
Sirt2 RNAi	2	Bloomington	36868
Sirt2 RNAi	3	Bloomington	31613
Sirt4 RNAi	1	VDRC	KK110639
Sirt4 RNAi	2	Bloomington	31638
Sirt4 RNAi	3	Bloomington	36588
Sirt4 RNAi	4	Bloomington	33984
Sirt6 RNAi	1	Bloomington	34530
Sirt6 RNAi	2	Bloomington	31399
Sirt6 RNAi	3	Bloomington	36801
Sirt7 RNAi	1	Bloomington	32483
Sirt7 RNAi	2	Bloomington	31093
Sirt7 RNAi	3	Bloomington	36802
AFG;;UAS-GFP		Cohen lab	
;enG4		Cohen lab	
HisC/Cyo-lacZ		Alf Herzig	
3xHis-GU H3R18, H4R8 (VK33)/TM6b		this study	
3xHis-GU H3R18, H4R8 (ZH86Fb)/TM6b		this study	
3xHis-GU H3Q18, H4Q8 (VK33)/TM6b		this study	
3xHis-GU H3Q18, H4Q8 (ZH86Fb)/TM6b		this study	
3xHis-GU H3R18, H4R8 (VK33), 3xHis-GU H3R18, H4R8 (ZH86Fb)/TM6b		this study	
3xHis-GU H3Q18, H4Q8 (VK33), 3xHis-GU H3Q18, H4Q8 (ZH86Fb)/TM6b		this study	
hsFLP; sp;+/S-T		Cohen lab	
nos-phiC31;;VK33		Cohen lab	
nos-phiC31;;ZH86Fb		Bloomington	24749
;tshG4/Cyo		Fernando Casares	
;;tubulinG4/TM6b		Cohen lab	
;;tubulinG80ts		Cohen lab	
;;UAS-AcCoAS		Bloomington	20761
;UAS-GFP		Cohen lab	
;UAS-GFP; hhG4/TM6b		Cohen lab	
;;UAS-IFP		Bloomington	64182
;UAS-mitoGFP/Cyo		Bloomington	8842
;;UAS-reaper/TM6b		Cohen lab	
;;UAS-SypHer3s-dmito		this study	
w[1118]		Bloomington	3605

Suppl. Table 3: Genotypes for all figure panels

Figures	panel	genotype	treatment
**Main figures**			
1	c-e	w[1118]	-
	f	;tshG4,UAS-GFP;+/S-T	-
	g	;tshG4,UAS-GFP/+; UAS-reaper/tubG80ts	1 d 29C
	h-i	w[1118]	-
	k	hsFLP/AFG;;UAS-GFP/+	15-25 min at 33°C during mid L3
2	a-b	w[1118]	-
	c-d	hsFLP/AFG;koi RNAi/+;UAS-GFP/+	15-45 min at 35°C during early-mid L3
	e-h	;nej RNAi #1/+ ;hhG4, tubG80ts, UAS-IFP/+	1 d 29C
3	a-b	w[1118]	2 h explant cultures: A485 (20 μM), butyrate (20mM), trichostatin A (500 nM), panobinostat (100 nM)
	c-d	;;hhG4, tubG80ts,UAS-IFP/HDAC1 RNAi #1	2 d 29C, 1 h explant cultures: A485 (20 μM)
	f-g	;;hhG4, tubG80ts,UAS-IFP/HDAC1 RNAi #1	2 d 29C
4	a	w[1118]	-
	b	;nej RNAi #1/+ ;hhG4, tubG80ts, UAS-IFP/+	1 d 29C
	c	w[1118]	-
	d	UAS-GFP/nej RNAi #1; hhG4/+	-
	e-f	w[1118]	2 h explant cultures: 2-deoxy-glucose (20 mM), glutaminase inhibitor 968 (50 μM), 6-diazo-5-oxo-L-norleucin (500 μM), etomoxir (500 μM)
	g-h	w[1118]	2 h explant cultures: etomoxir (500 μM), octanoate (10 mM)
5	a-b	;UAS-mitoGFP/+; tubulinG4/+	1 h explant cultures: 2-deoxy-glucose (20 mM), glutaminase inhibitor 968 (50 μM), 6-diazo-5-oxo-L-norleucin (500 μM), etomoxir (500 μM)
	c-d	w[1118]	2 h explant cultures: acetate (10 mM), etomoxir (500 μM)
6	a-b	w[1118]	2 h explant cultures: acetate (10 mM)
	c-d	;UAS-GFP/+; hhG4/UAS-AcCoAS	-
	e-f	w[1118]	-
**ED figures**			
ED1	a-b	w[1118]	-
	c-d	w[1118]	stained at early, mid, and late L3
	e-g'	w[1118]	-
	h-h'	;tshG4,UAS-GFP/+; UAS-reaper/tubG80ts	1 d 29C
	i-j	hsFLP/AFG;Rok RNAi/+;UAS-GFP/+	30-45 min at 35°C during early-mid L3

ED2	a-a"	;UAS-GFP/Gcn5 RNAi; hhG4/+	-
	b-b"	;UAS-GFP/Elp3 RNAi; hhG4/+	-
	c-d	;UAS-GFP/nej RNAi #1; hhG4/+	-
	e-f	;nej RNAi #2/+ ;hhG4, tubG80ts, UAS-IFP/+	1 d 29C
	g	;tshG4, UAS-GFP	1 d 29C
	g'	;tshG4, UAS-GFP/nej RNAi #1	1 d 29C
	g"	;tshG4, UAS-GFP/nej RNAi #2	1 d 29C
	h-i	w[1118]	15-60 min explant cultures: A485 (20 µM)
ED3	a-b	w[1118]	2 h explant cultures: trichostatin A (500 nM), splitomycin (50 µM), nicotinamide (20 mM)
	c	w[1118]	-
	d	;;hhG4, tubG80ts,UAS-IFP/HDAC1 RNAi #1	1 d 29C
	e	;;hhG4, tubG80ts,UAS-IFP/HDAC1 RNAi #2	1 d 29C
ED4	a	w[1118]	-
	b-c	;nej RNAi #2/+ ;hhG4, tubG80ts, UAS-IFP/+	1 d 29C
	d	w[1118]	-
	e-f	;nej RNAi #2/+ ;hhG4, tubG80ts, UAS-IFP/+	1 d 29C
	g-h	;PDHa RNAi/+ ;hhG4, tubG80ts, UAS-IFP/+	1 d 29C
	i-j	;PDK RNAi/+ ;hhG4, tubG80ts, UAS-IFP/+	1 d 29C
	k-l	w[1118]	2 h explant cultures: etomoxir (50-500 µM)
	m-n	w[1118]	2 h explant cultures: etomoxir (500 µM), rotenone (10 µM)
	o-p	w[1118]	2 h explant cultures: rotenone (2.5-25 µM)
	q-r	;UAS-GFP/+; hhG4/HDAC1 RNAi #2	1 h explant cultures: etomoxir (500 µM)
ED5	a-f	w[1118]	2 h explant cultures: etomoxir (500 µM)
	h-i	;UAS-mitoGFP/+; tubulinG4/+	30 min explant cultures: FCCP (10 µM)
	j	;PDHa RNAi/+ ;hhG4, tubG80ts, UAS-IFP/+	1 d 29C, 30 min explant cultures
	k	;PDK RNAi/+ ;hhG4, tubG80ts, UAS-IFP/+	1 d 29C, 30 min explant cultures
	l	;;UAS-SypHer3s-dmito/tubulinG4	1 h explant cultures: FCCP (10 µM)
	m	;;UAS-SypHer3s-dmito/tubulinG4	1 h explant cultures: 2-deoxy-glucose (20 mM), glutaminase inhibitor 968 (50 µM), 6-diazo-5-oxo-L-norleucin (500 µM), etomoxir (500 µM)
	n	w[1118]	-
	o	;nej RNAi #2/+ ;hhG4, tubG80ts, UAS-IFP/+	1 d 29C, 30 min explant culture
	p	;nej RNAi #1/+ ;hhG4, tubG80ts, UAS-IFP/+	1 d 29C, 30 min explant cultures
	q-q'	;UAS-mitoGFP/+; tubulinG4/+	-
	r-s	hsFLP/AFG;UAS-mitoGFP/+;UAS-IFP	25 min at 33°C during mid L3
ED6	a	;UAS-mitoGFP/+; tubulinG4/+	30 min explant cultures

	c-d	;ATPCL RNAi/+ ;hhG4, tubG80ts, UAS-IFP/+	2 d 29C
	e-f	w[1118]	2 h explant cultures: citrate (10 mM), etomoxir (500 μM)
	g-h	w[1118]	2 h explant cultures: ACSS2 inhibitor (20 μM)
	i-j	w[1118]	2 h explant cultures: acetate (10 mM), etomoxir (500 μM), ACSS2 inhibitor (20 μM)
ED7	a-h	w[1118]	2 h explant cultures: acetate (10 mM)
ED8	a	;UAS-GFP/+; hhG4/UAS-AcCoAS	-
	b-e	w[1118]	-
	f-g	hsFLP/AFG;koi RNAi/+;UAS-GFP/+	45 min at 35°C during early L3
	h-i	hsFLP/AFG;Rok RNAi/+;UAS-GFP/+	45 min at 35°C during early L3
	j-l	w[1118]	2 h explant cultures: etomoxir (500 μM)
ED9	a-b	w[1118]	2 h explant cultures: etomoxir (500 μM)
	c-k	w[1118]	-
	c'-k'	;nej RNAi #1/+ ;hhG4, tubG80ts, UAS-IFP/+	1.5 d 29C
ED10	a	;enG4/nej RNAi #1	18C
	b	;enG4/nej RNAi #2	18C
	c	;enG4/+; HDAC1 RNAi #1/+	18C
	d	;enG4/+; HDAC1 RNAi #2/+	18C
	e-f	w[1118]	-
	g-g'	;enG4/+; UAS-IFP/+	18C
	h-h'	;enG4/nej RNAi #1	18C
	i-i'	;enG4/nej RNAi #2	18C
	j-j'	;enG4/+; HDAC1 RNAi #2/+	18C

Supplementary data	panel	genotype	treatment
1	a-b	w[1118]	2 h explant cultures: A485 (20 μM), splitomycin (50 μM), nicotinamide (20 mM)
	c-c''	;UAS-GFP/Sirt1 RNAi #1; hhG4/+	-
	d	;UAS-GFP/Sirt1 RNAi #2; hhG4/+	-
	e-e''	;UAS-GFP/Sirt2 RNAi #1; hhG4/+	-
	f-f''	;UAS-GFP/Sirt4 RNAi #1; hhG4/+	-
	g-g''	;UAS-GFP/+; hhG4/Sirt6 RNAi #1	-
	h-h''	;UAS-GFP/+; hhG4/Sirt7 RNAi #1	-
2	a-b	;UAS-GFP/+; hhG4/HDAC1 RNAi #1	-
	c-d	;UAS-GFP/+; hhG4/HDAC1 RNAi #2	-
	e-f	;UAS-GFP/+; hhG4/HDAC1 RNAi #3	-

	g-h	;UAS-GFP/HDAC1 RNAi #4; hhG4/+	-
3	a-a"	;UAS-GFP/HDAC3 RNAi #1; hhG4/+	-
	b	;UAS-GFP/HDAC3 RNAi #2; hhG4/+	-
	c	;UAS-GFP/+; hhG4/HDAC3 RNAi #3	-
	d-d"	;UAS-GFP/+; hhG4/HDAC4 RNAi #1	-
	e-e"	;UAS-GFP/HDAC6 RNAi #1; hhG4/+	-
	f-f"	;UAS-GFP/HDAC11 RNAi #1; hhG4/+	-

**Supplementary table**	**panel**	**genotype**	**treatment**
1		RNAi line is indicated in the table. For RNAi lines on chromosome 2: UAS-GFP/ RNAi; hhG4/+ . For RNAi lines on chromosome 3: UAS-GFP/+; hhG4/RNAi.	

Suppl. Table 4: Primary and secondary antibodies

The lpp (guinea pig) antibody was used in Figure 2a-b and ED Figure 1h-h'.
The lpp (#2, rabbit) antibody was used in ED Figure 1g-g'.
The H3K18ac #2 (Cell Signaling) and #3 (Active Motif) were only used in ED Figure 1b.
All other H3K18ac stainings were performed using the antibody from Abcam.

AcCoAS: acetyl-CoA synthase
ac-K: total acetylated lysine
ATPCL: ATP citrate lyase
H3K18crot: H3K18 crotonylation
lpp: lipoprotein
PDH: pyruvate dehydrogenase
tubK40ac: α-tubulin lysine 40 acetylation

antibody	source species	source	product number	LOT	dilution
**Primary antibodies**					
AcCoAS/ACSS2	rabbit	Abcam	Ab264391	GR3350114-1	1:200
ac-K	rabbit	Cell Signaling	9814S	5	1:200
ATPCL/ACLY	rabbit	Novus Biologicals	NBP1-90266	R09359	1:200
H3	rabbit	Cell Signaling	2650S	3, 4	1:200
H3K9ac	rabbit	Active Motif	39137	9811002	1:500
H3K9me1	rabbit	Abcam	Ab9045	GR323589-1	1:500
H3K9me2	mouse	Abcam	Ab1220	GR183500-3	1:500
H3K9me3	rabbit	Abcam	Ab8898	GR3176468-1	1:500
H3K18ac	rabbit	Abcam	Ab1191	GR300534-1, GR3287957-1	1:500
H3K18ac #2	rabbit	Cell Signaling	9675	2	1:500
H3K18ac #3	rabbit	Active Motif	ACM-39756	14722003	1:500
H3K18crot	rabbit	Cusabio	CSB-PA010 418OA18crHU	G0822A	1:100
H3K27ac	rabbit	Abcam	Ab4729	GR323154	1:500
H3K36me3	rabbit	Abcam	Ab9050	GR166781-1	1:500
H4K8ac	rabbit	Abcam	Ab15823	GR3209076-1, GR3294416-1	1:500
HDAC1	rabbit	ProteinTech	10197-1-AP	106660	1:200
lpp	guinea pig	Eaton Lab [Eugster, 2007, https://doi.org/10.1016/j.devcel.2007.04.019]			1:100

lpp #2	rabbit	Eaton Lab [Eugster, 2007, https://doi.org/10.1016/j.devcel.2007.04.019]			1:100
nejire	guinea pig	Mannervik lab [Holmqvist, 2012, https://doi.org/10.1371/journal.pgen.1002769]			1:500
pH3 S10	mouse	Cell Signaling	9706S	17	1:200
pPDH S293	rabbit	Abcam	Ab92696	GR319281-1	1:200
tubK40ac	rabbit	Cell Signaling	5335	6	1:200
**Secondary antibodies**					
anti-rabbit TRITC	donkey	Jackson ImmunoResearch	711-025-152		1:250
anti-mouse TRITC	donkey	Jackson ImmunoResearch	715-025-150		1:250
anti-mouse FITC	donkey	Jackson ImmunoResearch	715-095-151		1:250
anti-guinea pig FITC	goat	Jackson ImmunoResearch	106-095-003		1:250
anti-guinea pig Cy5	donkey	Jackson ImmunoResearch	706-175-148		1:250

Suppl. Table 5: Primer sequences

purpose	target	orientation	sequence	notes
cloning	SypHer3s-dmito	forward	aaccgtcagatccagatctatgtccgtcct	BglII site
cloning	SypHer3s-dmito	reverse	gatatctcgagttaaaccgcctgttttaaaac	XhoI site
cloning	mutant histone	forward	tggaatatctggccgctgag	XhoI site
cloning	mutant histone	reverse	gtggtaaaggaggcagaggcttgggaaag	H4R8
cloning	mutant histone	forward	ctttcccaagcctctgcctcctttaccac	H4R8
cloning	mutant histone	reverse	gtggtaaaggaggccaaggcttgggaaag	H4Q8
cloning	mutant histone	forward	ctttcccaagccttggcctcctttaccac	H4Q8
cloning	mutant histone	reverse	tagtagccagttgtctgcgtggcgccttt	H3R18
cloning	mutant histone	forward	aaaggcgccacgcagacaactggctacta	H3R18
cloning	mutant histone	reverse	tagtagccagttgttggcgtggcgccttt	H3RQ18
cloning	mutant histone	forward	aaaggcgccacgccaacaactggctacta	H3RQ18
cloning	mutant histone	reverse	acacgcttggcatgaatgg	BstBI site
FISH	E5	forward	cgacgagtccttttgtgagc	length: 424 nt
FISH	E5	reverse	ccggtaatacgactcactatagggcggatgagggttggaggatt	
FISH	drm	forward	ctcttcaccgaccaatcagc	length: 305 nt
FISH	drm	reverse	ccggtaatacgactcactataggggcaaacattttgacaggcgg	
FISH	Gapdh2	forward	accaagaacactacccaccc	length: 200 nt
FISH	Gapdh2	reverse	ccggtaatacgactcactatagggtgtggctggtaattgaagagc	
FISH	grn	forward	acaacatggacacctctcga	length: 400 nt
FISH	grn	reverse	ccggtaatacgactcactatagggttcggtgccactgtcaattg	
FISH	hth	forward	catgcacggctaccattcg	length: 307 nt
FISH	hth	reverse	ccggtaatacgactcactataggggacttcgggatctgcggtat	
FISH	mamo	forward	tactacaagacgggagcctg	length: 305 nt
FISH	mamo	reverse	ccggtaatacgactcactatagggcagcgtcgtattctggttcg	
FISH	mirr	forward	ataaccacccagagctaccg	length: 350 nt
FISH	mirr	reverse	ccggtaatacgactcactatagggctgtcctccactcgatccac	
FISH	nej (probe 1)	forward	ccaaatctgcgtactctggc	length: 307 nt
FISH	nej (probe 1)	reverse	ccggtaatacgactcactatagggtttggctcggtatgggaact	
FISH	nej (probe 2)	forward	gcggtgctgtggacataaaa	length: 368 nt
FISH	nej (probe 2)	reverse	ccggtaatacgactcactatagggatagacccgcgatgttttgc	
FISH	Sox15	forward	tgggatgcaatacgaggact	length: 351 nt
FISH	Sox15	reverse	ccggtaatacgactcactatagggccctcgtatccgccatagtt	
FISH	zfh2	forward	tacaatcttgatggcagcgc	length: 431 nt
FISH	zfh2	reverse	ccggtaatacgactcactatagggagtcggtgttcagtgtttgc	

Suppl. Table 6: Inhibitors & other small compounds

compound	company	product number	solvent
ACSS2 inhibitor	Biozol	SEL-S8588	DMSO
A485	Cayman	Cay24119-1	DMSO
carbonyl cyanide-p-trifluoromethoxyphenylhydrazone (FCCP)	Biomol	Cay15218-10	ethanol
etomoxir	Sigma-Aldrich	E1905	H2O
glutaminase inhibitor 968 (968)	Sigma-Aldrich	SML1327	DMSO
nicotinamide (NAM)	Sigma-Aldrich	72340	H2O
panobinostat (PB)	Cayman	Cay13280-5	DMSO
rotenone	Sigma-Aldrich	45656	ethanol
sodium acetate	Roth	6773.2	H2O
sodium butyrate	AlfaAesar	A11079	H2O
sodium citrate	MP	199635	H2O
sodium octanoate	Sigma-Aldrich	C5038	H2O
splitomicin (split)	Biotrend	10-2615-5	DMSO
trichostatin A (TSA)	Sigma-Aldrich	T1952	DMSO
2-deoxy-glucose (2DG)	Sigma-Aldrich	D8375	H2O
6-diazo-5-oxo-L-norleucin (DON)	Sigma-Aldrich	D2141	H2O
