# Supplementary material for: Nuclear position and local acetyl-CoA production regulate chromatin state
Source: Nature. 2024 Jun 5;630(8016):466–74. doi: 10.1038/s41586-024-07471-4 (PMC11168921; doi:10.1038/s41586-024-07471-4)
Supplement: Supplementary file 2 — Reporting Summary [file 41586_2024_7471_MOESM2_ESM.pdf]

nature portfolio

Corresponding author(s): Aurelio Teleman

Last updated by author(s): Apr 10, 2024

Reporting Summary

Nature Portfolio wishes to improve the reproducibility of the work that we publish. This form provides structure for consistency and transparency in reporting. For further information on Nature Portfolio policies, see our Editorial Policies and the Editorial Policy Checklist.

Statistics

For all statistical analyses, confirm that the following items are present in the figure legend, table legend, main text, or Methods section.

n/a	Confirmed	
☐	☒	The exact sample size (*n*) for each experimental group/condition, given as a discrete number and unit of measurement
☐	☒	A statement on whether measurements were taken from distinct samples or whether the same sample was measured repeatedly
☐	☒	The statistical test(s) used AND whether they are one- or two-sided<br>*Only common tests should be described solely by name; describe more complex techniques in the Methods section.*
☒	☐	A description of all covariates tested
☐	☒	A description of any assumptions or corrections, such as tests of normality and adjustment for multiple comparisons
☐	☒	A full description of the statistical parameters including central tendency (e.g. means) or other basic estimates (e.g. regression coefficient) AND variation (e.g. standard deviation) or associated estimates of uncertainty (e.g. confidence intervals)
☐	☒	For null hypothesis testing, the test statistic (e.g. *F*, *t*, *r*) with confidence intervals, effect sizes, degrees of freedom and *P* value noted<br>*Give P values as exact values whenever suitable.*
☒	☐	For Bayesian analysis, information on the choice of priors and Markov chain Monte Carlo settings
☒	☐	For hierarchical and complex designs, identification of the appropriate level for tests and full reporting of outcomes
☐	☒	Estimates of effect sizes (e.g. Cohen's *d*, Pearson's *r*), indicating how they were calculated

Our web collection on statistics for biologists contains articles on many of the points above.

Software and code

Policy information about availability of computer code

Data collection	The software running the Leica confocal microscope was Leica Application Suite X (LAS X) version 3.5.2.18963, which is commercially available.
Data analysis	All software used in this study is publicly or commercially available:<br>Images were analyzed with FIJI.<br>Cut&Run sequencing data sets were analyzed using the galaxy server platform (https://usegalaxy.org)<br>- Adapter sequences were trimmed using Trim Galore! (version 0.6.3) (https://github.com/FelixKrueger/TrimGalore)<br>- Reads were aligned using Bowtie2 (version 2.4.2).<br>- Read duplicates were removed using MarkDuplicates (version 2.18.2.2) (http://broadinstitute.github.io/picard/).<br>- Peak calling was performed by MACS2 callpeak (version 2.1.1.20160309.6)<br>- Differential binding was evaluated using DiffBind (version 2.10.0)<br>- Peaks were annotated by ChIPseeker (version 1.18.0) using the genome annotation file from Ensembl (dm6, genes and gene prediction).<br>GO enrichment analysis was performed with the online tool http://www.webgestalt.org.<br>Figure preparation: Affinity Photo and Affinity Designer.<br>Data analysis: Microsoft Excel or Graphpad Prism.

For manuscripts utilizing custom algorithms or software that are central to the research but not yet described in published literature, software must be made available to editors and reviewers. We strongly encourage code deposition in a community repository (e.g. GitHub). See the Nature Portfolio guidelines for submitting code & software for further information.

Data

Policy information about availability of data

All manuscripts must include a data availability statement. This statement should provide the following information, where applicable:

- Accession codes, unique identifiers, or web links for publicly available datasets
- A description of any restrictions on data availability
- For clinical datasets or third party data, please ensure that the statement adheres to our policy

All deep sequencing datasets are available at NCBI Geo (accession GSE207486). Reviewer token: irklausybhoztqx

Human research participants

Policy information about studies involving human research participants and Sex and Gender in Research.

Reporting on sex and gender	Not applicable.
Population characteristics	Not applicable.
Recruitment	Not applicable.
Ethics oversight	Not applicable.

Note that full information on the approval of the study protocol must also be provided in the manuscript.

Field-specific reporting

Please select the one below that is the best fit for your research. If you are not sure, read the appropriate sections before making your selection.

☒ Life sciences ☐ Behavioural & social sciences ☐ Ecological, evolutionary & environmental sciences

For a reference copy of the document with all sections, see nature.com/documents/nr-reporting-summary-flat.pdf

Life sciences study design

All studies must disclose on these points even when the disclosure is negative.

Sample size	No sample size calculations were performed. Sample sizes were determined based on practical considerations (e.g. number of animals available, processing time required) and expected variations between animals for a given sample. Based on these considerations, sample sizes were set at >6 animals per genotype/condition, which allowed modest standard deviations and significant p values to be obtained.
Data exclusions	One outlier (as determined by ROUT) was removed from ED Fig. 10b'. Otherwise, no data were excluded.
Replication	Except where noted in the figure legends, experiments were replicated at least 2 times. Only data that reproduced are included in this manuscript.
Randomization	All animals were randomly alloted.
Blinding	The experiments were not blinded because they each contained an internal control and because blinding is not usual in this field.

Reporting for specific materials, systems and methods

We require information from authors about some types of materials, experimental systems and methods used in many studies. Here, indicate whether each material, system or method listed is relevant to your study. If you are not sure if a list item applies to your research, read the appropriate section before selecting a response.

Materials & experimental systems

n/a	Involved in the study
☐	☒ Antibodies
☒	☐ Eukaryotic cell lines
☒	☐ Palaeontology and archaeology
☐	☒ Animals and other organisms
☒	☐ Clinical data
☒	☐ Dual use research of concern

Methods

n/a	Involved in the study
☐	☒ ChIP-seq
☒	☐ Flow cytometry
☒	☐ MRI-based neuroimaging

Antibodies

Antibodies used

antibody source species source product number LOT
AcCoAS/ACSS2 rabbit Abcam Ab264391 GR3350114-1
ac-K rabbit Cell Signaling 9814S 5
ATPCL/ACLY rabbit Novus Biologicals NBP1-90266 R09359
H3 rabbit Cell Signaling 2650S 3, 4
H3K9ac rabbit Active Motif 39137 9811002
H3K9me1 rabbit Abcam Ab9045 GR323589-1
H3K9me2 mouse Abcam Ab1220 GR183500-3
H3K9me3 rabbit Abcam Ab8898 GR3176468-1
H3K18ac rabbit Abcam Ab1191 GR300534-1, GR3287957-1
H3K18ac #2 rabbit Cell Signaling 9675 2
H3K18ac #3 rabbit Active Motif ACM-39756 14722003
H3K18crot rabbit Cusabio CSB-PA010 418OA18crHU G0822A
H3K27ac rabbit Abcam Ab4729 GR323154
H3K36me3 rabbit Abcam Ab9050 GR166781-1
H4K8ac rabbit Abcam Ab15823 GR3209076-1, GR3294416-1
HDAC1 rabbit ProteinTech 10197-1-AP 106660
lpp guinea pig Eaton Lab [Eugster, 2007, https://doi.org/10.1016/j.devcel.2007.04.019]
lpp #2 rabbit Eaton Lab [Eugster, 2007, https://doi.org/10.1016/j.devcel.2007.04.019]
nejire guinea pig Mannervik lab [Holmqvist, 2012, https://doi.org/10.1371/journal.pgen.1002769]
pH3 S10 mouse Cell Signaling 9706S 17
pPDH S293 rabbit Abcam Ab92696 GR319281-1
tubK40ac rabbit Cell Signaling 5335 6
anti-rabbit TRITC donkey Jackson ImmunoResearch 711-025-152
anti-mouse TRITC donkey Jackson ImmunoResearch 715-025-150
anti-mouse FITC donkey Jackson ImmunoResearch 715-095-151
anti-guinea pig FITC goat Jackson ImmunoResearch 106-095-003
anti-guinea pig Cy5 donkey Jackson ImmunoResearch 706-175-148

Validation

The lpp antibodies from the laboratory of Suzanne Eaton were validated in the following publication:
PMID: 17609110 and the nejire antibody from the laboratory of Mattias Mannervik was validated in the following publication: PMID: 22737084.

The following antibodies were validated in this study by gene knockdown or over-expression: AcCoAs/ACSS2, ATPCL/ACLY, HDAC1, nejire, pPDH S293.

AcCoAS/ACSS2, Abcam, Ab264391: validated https://www.abcam.com/products/primary-antibodies/acss2-antibody-ab264391.html
ac-K, Cell Signaling, 9814S: validated https://www.cellsignal.com/products/primary-antibodies/acetylated-lysine-ac-k-2-100-multimab-rabbit-mab-mix/9814
ATPCL/ACLY, Novus Biologicals, NBP1-90266 : validated https://www.novusbio.com/products/atp-citrate-lyase-antibody_nbp1-90266
H3, Cell Signaling, 2650S: validated https://www.cellsignal.com/products/primary-antibodies/histone-h3-antibody-chip-formulated/2650
H3K9ac, Active Motif ,39137: validated https://www.activemotif.com/catalog/details/39137/histone-h3-acetyl-lys9-antibody-pab
H3K9me1, Abcam, Ab9045: validated https://www.abcam.com/products/primary-antibodies/histone-h3-mono-methyl-k9-antibody-chip-grade-ab9045.html
H3K9me2, Abcam, Ab1220: validated https://www.abcam.com/products/primary-antibodies/histone-h3-di-methyl-k9-antibody-mabcam-1220-chip-grade-ab1220.html
H3K9me3, Abcam, Ab8898: validated https://www.abcam.com/products/primary-antibodies/histone-h3-tri-methyl-k9-antibody-chip-grade-ab8898.html
H3K18ac, Abcam, Ab1191: validated https://www.abcam.com/products/primary-antibodies/histone-h3-acetyl-k18-antibody-chip-grade-ab1191.html
H3K18ac #2, Cell Signaling, 9675: validated https://www.cellsignal.com/products/primary-antibodies/acetyl-histone-h3-lys18-antibody/9675
H3K18ac #3, Active Motif, ACM-39756: validated https://www.activemotif.com/catalog/details/39755/histone-h3-acetyl-lys18-antibody-pab-3
H3K18crot, Cusabio, CSB-PA010 418OA18crHU: validated https://www.cusabio.com/Polyclonal-Antibody/Crotonyl-HIST1H3A--K18--Antibody-12784039.html
H3K27ac, Abcam, Ab4729: validated https://www.abcam.com/products/primary-antibodies/histone-h3-acetyl-k27-antibody-chip-

grade-ab4729.html
H3K36me3, Abcam, Ab9050: validated https://www.abcam.com/products/primary-antibodies/histone-h3-tri-methyl-k36-antibody-chip-grade-ab9050.html
H4K8ac, Abcam, Ab15823: validated https://www.abcam.com/products/primary-antibodies/histone-h4-acetyl-k8-antibody-chip-grade-ab15823.html
HDAC1, ProteinTech, 10197-1-AP: validated https://www.ptglab.com/products/HDAC1-Antibody-10197-1-AP.htm
pH3 S10, Cell Signaling, 9706S: validated https://www.cellsignal.com/products/primary-antibodies/phospho-histone-h3-ser10-6g3-mouse-mab/9706
pPDH S293, Abcam, Ab92696: validated https://www.abcam.com/products/primary-antibodies/pdha1-phospho-s293-antibody-ab92696.html
tubK40ac, Cell Signaling, 5335: validated https://www.cellsignal.com/products/primary-antibodies/acetyl-a-tubulin-lys40-d20g3-xp-174-rabbit-mab/5335

Animals and other research organisms

Policy information about studies involving animals; ARRIVE guidelines recommended for reporting animal research, and Sex and Gender in Research

Laboratory animals	Species: Drosophila melanogaster<br>Strains: Full genotype for each figure panel is provided in Suppl. Table 3.<br>Age: wL3
Wild animals	This study did not involve wild animals.
Reporting on sex	Sex was generally not considered in the study design - both males and females were analyzed together. Only for the analysis of adult wings in ED Figure 10g-j' are wings from males and females shown separately.
Field-collected samples	This study did not involve samples collected from the field.
Ethics oversight	This study does not require an ethical approval.

Note that full information on the approval of the study protocol must also be provided in the manuscript.

ChIP-seq

Data deposition

☒ Confirm that both raw and final processed data have been deposited in a public database such as GEO.

☒ Confirm that you have deposited or provided access to graph files (e.g. BED files) for the called peaks.

Data access links<br>*May remain private before publication.*	https://www.ncbi.nlm.nih.gov/geo/query/acc.cgi?acc=GSE207486
Files in database submission	GSM6290457 (wing disc control)<br>GSM6290458 (wing disc control 2h BR1)<br>GSM6290459 (wing disc control 2h BR2)<br>GSM6290460 (wing disc + etomoxir BR1)<br>GSM6290461 (wing disc + etomoxir BR2)<br>GSE207486_Galaxy24_ChIPseeker... (peak calling)
Genome browser session<br>(e.g. UCSC)	http://genome.ucsc.edu/s/willnow/CUT%26RUN_submission<br>note: control 2h BR2 was split into 2 tracks due to size.

Methodology

Replicates	control: 3 biological replicates<br>treatment: 2 biological replicates
Sequencing depth	control: total reads = 61 Mio, unique = 5.2 Mio<br>control 2h BR1: total reads = 45 Mio, unique = 4.3 Mio<br>control 2h BR2: total reads = 67 Mio, unique = 47.6 Mio<br>etomoxir BR1: total reads = 57 Mio, unique = 4.7 Mio<br>etomoxir BR2: total reads = 72 Mio, unique = 4.3 Mio<br><br>reads: paired-end<br>sequence length: 101 bp
Antibodies	H3K18ac rabbit Abcam Ab1191 GR300534-1, GR3287957-1

Peak calling parameters	Peak calling was performed using the galaxy server plattform using MACS2 callpeak (version 2.1.1.20160309.6, standard setting).
Data quality	Read duplicates were removed using the galaxy server plattform using MarkDuplicates (version 2.18.2.2) . Peak calling used a FDR cut off of 5%.
Software	The galaxy server platform 25 was used for data analysis. Adapter sequences were trimmed using Trim Galore! (version 0.6.3) (https://github.com/FelixKrueger/TrimGalore) before aligning read sequences to the Drosophila genome (dm6) by Bowtie2 (version 2.4.2). Bowtie2 settings were adjusted according to Skene et al. as follows: --local --very-sensitive-local --no-unal --no-mixed --no-discordant --phred33 -I 10 -X 700. Next, read duplicates were removed using MarkDuplicates (version 2.18.2.2) (http://broadinstitute.github.io/picard/). Peak calling was performed by MACS2 callpeak (version 2.1.1.20160309.6). Finally, differential binding was evaluated using DiffBind (version 2.10.0) by grouping the samples according to treatment. Peaks were annotated by ChIPseeker (version 1.18.0) using the genome annotation file from Ensembl (dm6, genes and gene prediction).
